# Evaluation of mammographic density patterns: reproducibility and concordance among scales

**DOI:** 10.1186/1471-2407-10-485

**Published:** 2010-09-13

**Authors:** Macarena Garrido-Estepa, Francisco Ruiz-Perales, Josefa Miranda, Nieves Ascunce, Isabel González-Román, Carmen Sánchez-Contador, Carmen Santamariña, Pilar Moreo, Carmen Vidal, Mercé Peris, María P Moreno, Jose A Váquez-Carrete, Francisca Collado-García, Francisco Casanova, María Ederra, Dolores Salas, Marina Pollán

**Affiliations:** 1 National Centre for Epidemiology, Instituto de Salud Carlos III, Madrid, Spain; 2 Valencia Breast Cancer Screening Programme, General Directorate Public Health, Valencia, Spain; 3 Centro Superior de Investigación en Salud Pública(CSISP), Valencia, Spain; 4 Navarra Breast Cancer Screening Programme, Public Health Institute, Pamplona, Spain; 5 Consortium for Biomedical Research in Epidemiology & Public Health (CIBER en Epidemiología y Salud Pública - CIBERESP), Spain; 6 Castilla-Leon Breast Cancer Screening Programme, D.G. Salud Pública ID e I, SACYL, Castilla y León, Spain; 7 Balearic Islands Breast Cancer Screening Programme, Health Promotion for Women and Childhood, General Directorate Public Health and Participation, Regional Authority of Health and Consumer Affairs, Balearic Islands, Spain; 8 Galicia Breast Cancer Screening Programme, Regional Authority of Health, Galicia Regional Government, Spain; 9 Aragon Breast Cancer Screening Programme, Health Service of Aragon, Zaragoza, Spain; 10 Cancer Prevention and Control Unit, Catalan Institute of Oncology (ICO), Barcelona, Spain

## Abstract

**Background:**

Increased mammographic breast density is a moderate risk factor for breast cancer. Different scales have been proposed for classifying mammographic density. This study sought to assess intra-rater agreement for the most widely used scales (Wolfe, Tabár, BI-RADS and Boyd) and compare them in terms of classifying mammograms as high- or low-density.

**Methods:**

The study covered 3572 mammograms drawn from women included in the DDM-Spain study, carried-out in seven Spanish Autonomous Regions. Each mammogram was read by an expert radiologist and classified using the Wolfe, Tabár, BI-RADS and Boyd scales. In addition, 375 mammograms randomly selected were read a second time to estimate intra-rater agreement for each scale using the kappa statistic. Owing to the ordinal nature of the scales, weighted kappa was computed. The entire set of mammograms (3572) was used to calculate agreement among the different scales in classifying high/low-density patterns, with the kappa statistic being computed on a pair-wise basis. High density was defined as follows: percentage of dense tissue greater than 50% for the Boyd, "heterogeneously dense and extremely dense" categories for the BI-RADS, categories P2 and DY for the Wolfe, and categories IV and V for the Tabár scales.

**Results:**

There was good agreement between the first and second reading, with weighted kappa values of 0.84 for Wolfe, 0.71 for Tabár, 0.90 for BI-RADS, and 0.92 for Boyd scale. Furthermore, there was substantial agreement among the different scales in classifying high- versus low-density patterns. Agreement was almost perfect between the quantitative scales, Boyd and BI-RADS, and good for those based on the observed pattern, i.e., Tabár and Wolfe (kappa 0.81). Agreement was lower when comparing a pattern-based (Wolfe or Tabár) versus a quantitative-based (BI-RADS or Boyd) scale. Moreover, the Wolfe and Tabár scales classified more mammograms in the high-risk group, 46.61 and 37.32% respectively, while this percentage was lower for the quantitative scales (21.89% for BI-RADS and 21.86% for Boyd).

**Conclusions:**

Visual scales of mammographic density show a high reproducibility when appropriate training is provided. Their ability to distinguish between high and low risk render them useful for routine use by breast cancer screening programs. Quantitative-based scales are more specific than pattern-based scales in classifying populations in the high-risk group.

## Background

Increased mammographic breast density is a moderate independent risk factor for breast cancer, ranking only behind age and family history of breast cancer[1,2]. Different studies have reported an attributable risk of around 30% for mammographic densities of over 50%[3,4], when classical risk factors, taken together, explain less than 50% of the overall incidence. Although different classifications have been used, the odds ratio for developing breast cancer for the most compared with the least dense breast tissue categories ranges from 1.8 to 6.0, with most studies yielding an odds ratio of 4.0 or greater[5]. Recently, measurement of breast density has been proposed as an intermediate phenotype for breast cancer, useful in epidemiologic, clinical and genetic studies[2]. The first classification was introduced by Wolfe in 1976[6], based on qualitative and quantitative criteria of breast parenchyma to describe four different patterns. Attempts to increase reproducibility entailed the creation of quantitative methods, the most widely used being that proposed by Boyd, which uses a semi-quantitative score of six categories[4]. In 1997, Tabár proposed a modification of Wolfe's classification, with five categories based on anatomic-mammographic correlations[7]. Another classification, Breast Imaging Reporting and Data System (BI-RADS), was developed in the USA to standardize mammography reports, reduce confusion in the interpretation of breast images, and facilitate the monitoring of results.

First edition of BI-RADS classification described four categories of density patterns, but later 2003 edition define them based on quantitative criteria as a quartiles of density percentages [8,9]. At the end of the nineties computer-assisted measurements of breast density have been developed to calculate percentages of mammographic density but there are some limitations to the technique, such as the digitization of the images, time consumed, need for specific training, difficulty in mixing analog and digital mammograms, and the fact that such techniques continue to be non-volumetric[10,11]. At present, this computer-assisted method is not routinely used in radiology units, and has not been validated for digital mammograms.

This present study sought to: assess the intra-rater reproducibility of the different visual scales of classification; and compare agreement among the Wolfe, Tabár, BI-RADS and Boyd scales in terms of classification into high- versus low-density groups. Lastly, we explored the variability of the Wolfe and Tabár qualitative pattern-based classifications with respect to the six categories of the Boyd semiquantitative scale.

## Methods

### Subjects and mammograms

We used data drawn from the "Determinants of Density in Mammography in Spain" (DDM-Spain) study. This was a cross-sectional study which aimed to identify genetic, reproductive and lifestyle characteristics associated with mammographic patterns/densities that might enhance the risk of developing breast cancer. Briefly, women aged 45 and over who attended the regional Breast Cancer Screening Programs at the recruiting centers established in Barcelona, Burgos, Corunna (*Coruña*), Palma de Mallorca, Pamplona, Valencia and Zaragoza from September 2006 through June 2007 were invited to participate in the study. Exclusion criteria of DDM-Spain included the following: women not born in Spain; evidence of previous breast or ovarian cancer; inability to answer the questionnaire; physical impairment to perform the mammogram; and previous breast surgery or implants. The study was reviewed and approved by the Bioethics Committee of the Instituto de Salud Carlos III (Madrid) and all subjects provided written consent. The intended sample size was 500 women per center, implying a total of 3500 women. The final sample consisted of 3572 women (range 496 to 534 per center). The average participation rate was 74.5%, ranging from 64.7% in Corunna to 84.0% in Zaragoza. Mammogram quality at each of these centers had been explored by a pilot study using 25 mammograms per center. As the cranio-caudal (CC) projection posed fewer technical problems, we decided to evaluate mammographic density using the CC projection from the left breast.

Of the total included in the study, 2040 mammograms were in analog format (those from Burgos, Corunna, Pamplona and Zaragoza) and the remaining 1532 were in digital format (those from Barcelona, Palma de Mallorca and Valencia).

### Measurements

Mammographic density was evaluated by a single experienced radiologist (Dr. Francisco Ruíz-Perales). All mammograms were classified using the Wolfe, Tabár, BI-RADS and Boyd scales: analog mammograms were read in negatoscope (view box), and digital mammograms on a computer screen. Every mammogram (in total 3572) was read randomly in four different stages to prevent recall bias until every scale was completed to study comparability among scales.

To obtain a subsample of twice-read mammograms to explore intra-observer agreement each of the participant centers chose a set of 50-60 consecutive images using a random number between the first and last mammograms as initial. If the random number proposed was too near the last, the reading continued from the first. The second reading of a total of 375 mammograms was performed with the same procedure than first beginning within 1 to 66 days after the end of the previous reading to prevent recall bias.

### Statistical methods

Intra-observer agreement was evaluated using the kappa coefficient, since it requires no assumption about correct categorization and includes correction for the degree of agreement that would be expected by chance alone[12]. Due to the ordinal nature of the scales, weighted kappa was also calculated.

Wolfe scale is compound of four categories defined as N1, P1, P2 and DY while Tabár proposed five different patterns from I to V (See table 1 and table 2 for further description of categories).

**Table 1:** Agreement between the first and second measures using the Wolfe classification

Number of mammograms classified in each category in the first and second measurements					
	**Second measure**				
**First measure**	*N1*	*P1*	*P2*	*DY*	*Total*
*N1*	12	9	0	0	21
*P1*	4	139	13	5	161
*P2*	0	7	101	14	122
*DY*	0	2	13	56	71
*Total*	16	157	127	75	375
**Kappa-statistic estimation**					
*Agreement*		*Kappa (95% CI)*	*Weighted Kappa (95% CI)*	*Kappa high/low risk (95% CI)*	
*Observed %*	*Expected %*				
82.13	33.02	0.733 (0.674-0.789)	0.835 (0.784-0.876)	0.851 (0.798-0.904)	

Low risk categories:

N1: Breast composed almost completely of fat, with perhaps just a few fibrous connective tissue strands.

P1: Breast composed mainly of fat, although up to a quarter of the sub-areolar area may show beaded or cord-like areas corresponding to prominent ducts.

High risk categories:

P2: More severe involvement of the breast, with a prominent duct pattern occupying more than one quarter of breast volume.

DY: Breast typically contains extensive regions of homogeneous mammographic densities, which appear as sheet-like regions. The proportion of density is greater than that of the fat.

**Table 2:** Agreement between the first and second measures using the Tabár classification

Number of mammograms classified in each category in the first and second measurements					
	**Second measure**				
**First measure**	*II*	*III*	*IV*	*V*	*Total*
*II*	12	9	0	0	21
*III*	4	170	16	8	198
*IV*	0	4	114	6	124
*V*	0	8	9	15	32
*Total*	16	191	139	29	375
**Kappa-statistic estimation**					
*Agreement*		*Kappa (95% CI)*	*Weighted Kappa (95% CI)*	*Kappa high/low risk (95% CI)*	
*Observed %*	*Expected %*				
82.93	40.05	0.715 (0.649-0.772)	0.707 (0.623-0.781)	0.800 (0.739-0.861)	

Low risk categories:

I: Mammogram composed of scalloped contours with some lucent areas of fatty replacement and 1 mm evenly distributed nodular densities.

II: Mammogram composed almost entirely of lucent areas of fatty replacement and 1-mm evenly distributed nodular densities.

III: Prominent ducts in the retroareolar area.

High risk categories:

IV: Extensive, nodular and linear densities with nodular size larger than normal lobules.

V: Homogeneous ground-glass-like appearance with no perceptible features.

Quantitative based scales, BI-RADS and Boyd, are defined using percentages of density. BI-RADS into quartiles and Boyd divided into six categories of unequal intervals: "A" 0%; "B" > 0-10%; "C" > 10-25%; "D" > 25-50%; "E" > 50-75% and "F" > 75% (Table 3 and 4).

**Table 3:** Agreement between the first and second measures using the BI-RADS classification

Number of mammograms classified in each category in the first and second measurements					
	**Second measure**				
**First measure**	Almost entirely fat	Scattered fibroglandular densities	Heterogeneously dense	Extremely dense	Total
Almost entirely fat	147	13	0	0	160
Scattered fibroglandular densities	14	101	10	0	125
Heterogeneously dense	0	14	48	6	68
Extremely dense	0	0	3	19	22
Total	161	128	61	25	375
**Kappa-statistic estimation**					
*Agreement*		*Kappa (95% CI)*	*Weighted Kappa (95% CI)*	*Kappa high/low risk (95% CI)*	
*Observed %*	*Expected %*				
84.00	33.04	0.761 (0.706-0.814)	0.904 (0.877-0.928)	0.815 (0.746-0.885)	

Low risk categories:

Almost entirely fat: 0-25%

Scattered fibroglandular densities: > 25-50%

High risk categories:

Heterogeneously dense: > 50-75%

Extremely dense: > 75%

**Table 4:** Agreement between the first and second measures using the Boyd classification

Number of mammograms classified in each category in the first and second measurements							
	**Second measure**						
**First measure**	*A*	*B*	*C*	*D*	*E*	*F*	*Total*
*A = 0%*	6	4	0	0	0	0	10
B = > 0-10*%	4	56	11	0	0	0	71
C = 10-25*%	0	16	50	13	0	0	79
D = 25-50*%	0	0	14	102	9	0	125
E = 50-75*%	0	0	0	14	48	6	68
*F = *≥*75%*	0	0	0	0	3	19	22
*Total*	10	76	75	129	60	25	375
**Kappa-statistic estimation**							
*Agreement*		*Kappa (95% CI)*		*Weighted Kappa (95% CI)*		*Kappa high/low risk (95% CI)*	
*Observed %*	*Expected %*						
74.93	22.88	0.675 (0.616-0.733)		0.917 (0.898-0.933)		0.822 (0.754-0.891)	

Low risk categories:

A: 0%

B: > 0-10*%

C: 10-25*%

D: 25-50*%

High risk categories:

E: 50-75*%

F: ≥75%

*Upper bound excluded

To compare different classification scales having different numbers of categories, a dichotomous re-classification was defined for each scale, grouping different categories into low- and high-risk groups (Table 1, 2, 3 and 4). The Wolfe classification low-risk group was formed by categories N1 and P1 and the high-risk group by categories P2 and DY. For Tabár classification, I-III were considered low-risk and IV-V high-risk. BI-RADS was divided into low-risk (almost entirely fat and scattered fibroglandular densities) and high-risk (heterogeneously dense and extremely dense). Finally, in the Boyd classification, mammograms classified as A, B, C and D were included in the low-risk group, and E and F categories as high-risk.

High/low-risk classifications enabled agreement between the four scales to be compared, using percentage agreement and the kappa statistic. Comparison between the pattern-based classification of the Wolfe, Tabár scales and semi-quantitative scales, BI-RADS and Boyd, was studied graphically to observe how their respective categories expressed density percentages.

Confidence intervals were calculated. Bootstrapping methods were used when more than two categories or weighted kappa were involved; to optimize the results, bootstrapping was performed with 5000 replications. In high/low-risk categorization, confidence intervals for the kappa statistic were calculated using a previously described analytical method[13].

All statistical analyses were performed using the Stata version 10 computer software program.

## Results

### Intra-observer agreement

#### Wolfe's classification

Table 1 shows agreement between the first and second measurements using the Wolfe scale, with 308 of the 375 (82.13%) images being consistently classified. Only 1.86% of the observations showed disagreement in two categories. The kappa value was 0.73 (p < 0.0001) and the weighted kappa value was 0.84 (p < 0.0001). Using aggregated data in two categories (low- and high-risk), the kappa statistic was 0.85 (p < 0.0001).

Kappa values for analog and digital measures were 0.78 (p < 0.0001, 95% CI: 0.706-0.846) and 0.67 (p < 0.0001, 95% CI: 0.575-0.764), and weighted kappa values were 00.87 (p < 0.0001, 95% CI: 0.808-0.916) and 0.78 (p < 0.0001, 95% CI: 0.686-0.856), respectively

#### Tabár's classification

Comparisons between first and second measures with Tabár classification are shown in Table 2. The percentage of total agreement was 82.93% (311 of 375 measures were exactly the same). Only 4.27% of the measures differed in two categories. The kappa value was 0.72 (p < 0.0001) and that of weighted kappa 0.71 (p < 0.0001). When the scale was divided into low and high risk, the kappa value was 0.80 (p < 0.0001)

Kappa values for analog and digital measures were 0.77 (p < 0.0001; 95% CI: 0.685-0.838) and 0.62 (p < 0.0001; 95% CI: 0.497-0.721), and weighted kappa values were 0.75 (p < 0.0001; 95% CI: 0.641-0.845) and 0.64 (p < 0.0001; 95% CI: 0.494-0.764), respectively.

#### BI-RADS classification

Using the BI-RADS scale, 315 of 375 images were classified in the same category (84% agreement). No observations differed in more than one category, as can be seen in Table 3. The kappa value for BI-RADS classification was 0.76 (p < 0.0001) and weighted kappa was 0.90 (p < 0.0001). High/low-risk classification resulted in a kappa value of 0.82 (p < 0.0001).

Kappa values for analog and digital measures were 0.76 (p < 0.0001; 95% CI: 0.683-0.829) and 0.76 (p < 0.0001; 95% CI: 0.676-0.842), and weighted kappa values were 0.90 (p < 0.0001; 95% CI: 0.864-0.932) and 0.90 (p < 0.0001; 95% CI: 0.860-0.938), respectively.

#### Boyd's classification

Table 4 summarizes the agreement between the first and second measure using the Boyd scale. Total agreement was 74.93% (281 of 375 measures were identically classified). There were no observations with disagreement in more than one category. The kappa value was 0.68 (p < 0.0001) and weighted kappa was 0.92 (p < 0.0001). Using the two-category scale, the kappa statistic was 0.82 (p < 0.0001).

Kappa values for analog and digital measures were 0.70 (p < 0.0001; 95% CI: 0.626-0.774) and 0.63 (p < 0.0001; 95% CI: 0.547-0.724), and weighted kappa values were 0.92 (p < 0.0001; 95% CI: 0.890-0.940) and 0.91 (p < 0.0001; 95% CI: 0.884-0.936), respectively

#### Comparability among scales

High/low-risk categorizations enabled the four scales to be compared. The bivariate study of interscale agreement is summarized in Table 5.

**Table 5:** Bivariate study for high/low risk classification among scales

Percentage of total agreement; Kappa statistics (95% CI)					
**Scale**		**Wolfe**	**Tabár**	**BI-RADS**	**Boyd**

Wolfe	% Agreement:	92.55%*	90.37%	75.22%	75.25%
	Kappa (95% CI):	0.851 (0.798-0.904)*	0.804 (0.785-0.823)	0.485 (0.460-0.510)	0.485 (0.460-0.511)
Tabár	% Agreement:	90.37%	90.16%*	84.46%	84.49%
	Kappa (95% CI):	0.804 (0.785-0.823)	0.800 (0.739-0.861)*	0.638 (0.612-0.663)	0.638 (0.612-0.664)
BI-RAD	% Agreement:	75.22%	84.46%	93.35% *	99.97%
	Kappa (95% CI):	0.485 (0.460-0.510)	0.638 (0.612-0.663)	0.815 (0.746-0.885)*	0.999 (0.998-1.000)
Boyd	% Agreement:	75.25%	84.49%	99.97%	93.62%*
	Kappa (95% CI):	0.485 (0.460-0.511)	0.638 (0.612-0.664)	0.999 (0.998-1.000)	0.822 (0.754-0.891)*

*Data drawn from intra-observer agreement study (sub-sample of 320 mammograms)

#### Wolfe's classification

Wolfe classified 53.39% of the mammograms as low-risk and 46.61% as high-risk. Total agreement between Wolfe's and the Boyd, BI-RADS and Tabár scales was 75.25%, 75.22% and 90.37% with kappa values of 0.49, 0.49 and 0.80, respectively.

#### Tabár's classification

Using Tabár's classification, 37.32% mammograms were classified as high-risk.

Whereas Tabár displayed almost perfect agreement with Wolfe (90.37% agreement and a kappa value of 0.80 (p < 0.00001)), agreement with BI-RADS and Boyd was lower (84.46% and 84.49%, respectively), with kappa values of 0.64 for both (p < 0.0001)

#### BI-RADS classification

BI-RADS classified 21.89% mammograms as high-risk. BI-RADS agreement with the other classifications was as follows: Wolfe, 75.22%, and a kappa value of 0.48 (p < 0.0001); Tabár, 84.46%, and a kappa value of 0.64; BI-RADS and Boyd, almost perfect (99.97% total agreement and a kappa value of almost 1.00, namely, 0.99992 with p < 0.0001).

#### Boyd's classification

Boyd classified 78.14% of mammograms as low-risk and 21.86% as high-risk. It showed almost perfect agreement with BI-RADS (99.97% total agreement and a kappa value of almost 1, namely, 0.9992, p < 0.0001), good agreement with Tabár (84.49% agreement and a kappa value of 0.64, p < 0.0001), and moderate agreement with Wolfe (75.25% agreement and a kappa value of 0.49, p < 0.0001).

#### Graphic distribution study of qualitative scales of mammographic density measurement (Wolfe, Tabár and BI-RADS) with respect to a semi-quantitative scale (Boyd)

**Figure 1:**
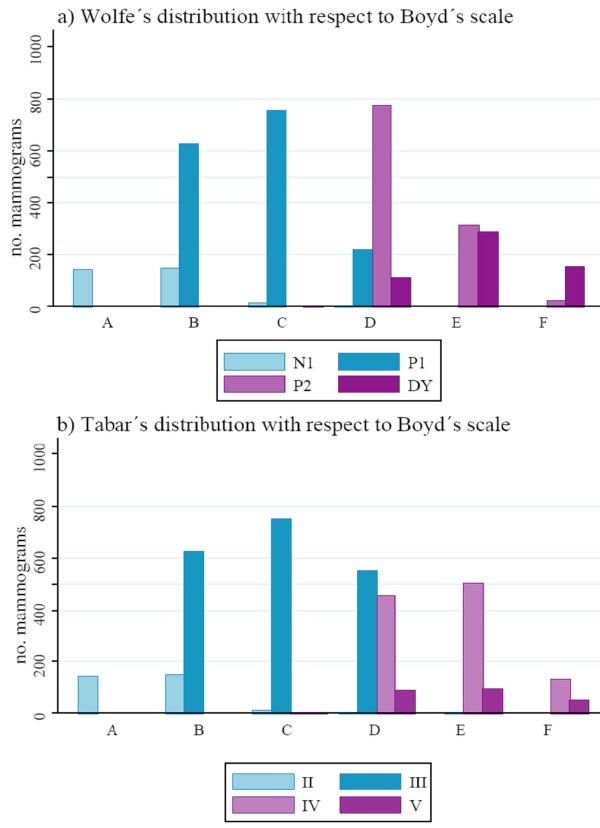
**Graphic distribution study of mammographic density measured by qualitative scales with respect to Boyd's low-risk (A, B, C, D) and high-risk categories (E, F)**.

Figure 1 shows the distribution of the different qualitative (i.e., pattern-based) scales of mammographic density measurement with respect to Boyd's scale. Low-risk categories are represented in blue and high-risk categories in violet-purple. Boyd categories A, B, C and D correspond to low risk, and E and F to high risk.

None of the mammograms of low-risk categories of the Wolfe scale, N1 and P1, were included in the high-risk categories of the Boyd scale (E and F) and only one mammogram classified as low risk, category III, using Tabár scale was included in the high risk category E of Boyd scale. Categories N1 (Wolfe) and II (Tabár) are classified mainly into categories A or B of Boyd scale (≤ 10% of density) and categories P1 and III into B, C or D (> 0-50%). On the other hand, 24.75% of the mammograms included in high-risk categories P2 and DY using Wolfe method, were included in low-risk categories, almost all in D category, using Boyd scale. For Tabár scale the percentage was lower, 15.48% (15.34% in D).

Category-D mammograms, namely, those with 25% to 50% density, displayed a more heterogeneous distribution under Wolfe's and Tabár's systems.

## Discussion

Different methods in use, subjective classifications, and the lack of a gold standard mean that misclassification is a potential problem when assessing mammographic density and potential breast cancer risk. Previous studies have measured inter- and intra-observer variability using experienced radiologists. Most of these studies report good or very good agreement[14-23]. Reliability studies of Wolfe's classification show kappa values ranging from 0.69 to 0.88 for inter-observer agreement, and 0.69 to 0.87 for intra-observer agreement[14-18]. In previous studies: Tabár inter-observer agreement obtained kappa values of 0.63 and of 0.75 for intra-observed agreement[19]; BI-RADS classification displayed only moderate agreement, with an overall kappa value for intra-observer agreement of 0.43-0.59[20,21]; and Boyd's classification registered inter-observer agreement of 0.89[22] and a kappa value of 0.74[18], with a weighted kappa value for intra-observer agreement of 0.68-0.89[19,23].

In our study, good intra-observer reproducibility was observed, particularly when weighted kappa was used. Only the Wolfe and Tabár scales showed disagreement in more than one category in 7 (1.86%) and 16 (4.27%) cases, respectively: six of these cases were classified on both scales with disagreement in two categories, perhaps due to specific characteristics of these mammograms which hindered their evaluation. Only one classification -Boyd's- registered a kappa value of under 0.70, namely, 0.68. Nevertheless, all the disagreements observed (25.06%) corresponded to differences in only one category. It should be noted that Boyd's classification is divided into six categories, with three categories -A, B and C- classifying densities under 25 percent with narrower range intervals than the rest. Half of all mammograms with different results in both readings belonged to these three categories. Taking into account the number of categories and the semi-quantitative nature of the Boyd scale, weighted kappa is a more appropriate estimator of concordance. Using this statistic, concordance for Boyd's scale was 0.92. The other classifications registered agreement percentages of 82% to 84%, and weighted kappa values of over 0.75. Previous studies using the BI-RADS scale reported moderate agreement, with kappa statistics of 0.43 to 0.59 for intra-observer studies[16,17]. We obtained kappa and weighted kappa values of 0.76 and 0.90 respectively, showing very good agreement.

When comparing the different scales, kappa values for distinguishing high-density mammographic patterns ranged from 0.79 to 0.86, revealing almost perfect agreement. This good correlation among the different scales explains the consistency of results on the relationship between mammographic density and breast cancer obtained from different studies using different scales[1,2]. It is interesting to note, however, that classifications, such as Tabár's and Wolfe's, which consider both qualitative and quantitative information on density, displayed lower concordance with the semi-quantitative scale. A more detailed analysis confirmed that these scales registered the greatest disagreement in mammograms in the intermediate dense-tissue percentage category, i.e., ranging from 25% to 50%. This means that categories associated to high risk of the Wolfe and Tabár scales are classify with a huge variability from 25 to 100% of density using quantitative based scales and some women are classified into low (< 50% of density) or high risk group depending on the method selected. It would have been interesting to ascertain to what extent qualitative information in such cases determined differences in breast cancer risk and the clinical relevance of classifying different population into high and low risk but our study was unable to address this issue directly.

Separate comparison between digital and analog images failed to reveal relevant differences, yielding weighted kappa values for analog versus digital of: 0.87 versus 0.78 using Wolfe's scale; 0.75 versus 0.64 using Tabár's scale; 0.90 versus 0.90 using the BI-RADS scale; and 0.92 versus 0.91 using Boyd's scale. Even though these differences did not attain statistical significance, the kappa values were always slightly higher when our reader examined analog images. This may reflect his longer experience with the old technology, since digital mammographic technology has only recently been introduced in Spanish screening programs.

Limitations are intra-observer design of the study and the lack of comparison with computer-assisted methods, which would result in more objective measurements of breast density, slightly higher agreement values, and the possibility of obtaining a measure of density percentages as a continuous variable. Furthermore, such methods are also dependent on observer experience, since the program has to be given some pointers to enable it to delimit the area in which it must calculate the percentage of the breast occupied by dense tissue[1,2]. This technique has not been introduced in Spanish breast cancer screening programs, and no radiologist or technician with the necessary experimental training could be found who was able to use it. Previous studies have shown excellent reproducibility, with an intraclass correlation of over 0.9[11] and a Pearson correlation with r values of over 0.90[10], when this method was used on previously digitized analog images.

## Conclusions

Visual classification of mammographic density patterns, besides being quick and easy to perform, is a relatively inexpensive method to implement in breast cancer screening programs. Our study confirms that, using an experienced reader, the four scales display very high reproducibility and are extremely consistent in identifying women with high-density patterns. Quantitative-based scales are more specific in classifying populations in the high-risk group.

## Abbreviations

BI-RADS: Breast Imaging Reporting and Data System; DDM-Spain study: Determinants of Density in Mammography in Spain

## Competing interests

The authors declare that they have no competing interests.

## Authors' contributions

MP was responsible for the study concept. NA, IGR, CSC, CS, PM, CV & DS were the main responsible of DDM-Study in the corresponding screening programs and provided valuable input to the study desing. FRP & JM conducted a pilot study to verify the quality of mammograms in the different programs. FRP evaluated mammographic density using the four scales. MG & MP performed the statistical analysis and prepared the first draft of the manuscript. The rest of the authors were involved in the field work of the study, recruiting participants, collecting the corresponding mammograms and the information needed to carry out the study. All authors critically revised the manuscript and suggested helpful comments that were subsequently introduced in the text. All authors read & approved the final manuscript.

## Pre-publication history

The pre-publication history for this paper can be accessed here:

http://www.biomedcentral.com/1471-2407/10/485/prepub
